# Management of Gastrointestinal Schwannomas: A State-of-the-Art Review

**DOI:** 10.1007/s12029-026-01479-x

**Published:** 2026-05-21

**Authors:** Daniel Paramythiotis, Dimitrios Tsavdaris, Konstantina Katsiafliaka, Niki Vasiliki Tsakiri, Panagiotis Tsintidis, Maria Angeliki Kreza, Elizabeth Psoma, Alexandros Mekras, Antonios Michalopoulos

**Affiliations:** 1 First Department of Propaedeutic Surgery, AHEPA University General Hospital of Thessaloniki, Aristotle University of Thessaloniki, Thessaloniki, Greece; 2 School of Medicine, Aristotle University of Thessaloniki, Thessaloniki, Greece; 3 Department of Clinical Radiology, University General Hospital of Thessaloniki AHEPA, Aristotle University of Thessaloniki, Thessaloniki, Greece

**Keywords:** Gastrointestinal schwannoma, Mesenchymal tumors, Subepithelial lesions, SOX10, S100, Differential diagnosis, Endoscopic resection, Minimally invasive surgery, Radiomics

## Abstract

**Introduction:**

Gastrointestinal schwannomas are rare mesenchymal tumors that arise from the Schwann cells of the enteric nervous system, representing a unique clinicopathological entity among subepithelial lesions of the gastrointestinal tract. The aim of this state-of-the-art review is therefore to analyze the specific clinicopathological characteristics of these rare tumors as well as to better understand the appropriate diagnostic tools and therapeutic approaches where these are required.

**Methods:**

This review combines findings from large series studies to provide a complete analysis of gastrointestinal schwannomas, even those with unusual locations.

**Results:**

Despite the typical occurrence of these tumors within the stomach, they can occur throughout the gastrointestinal tract, from the esophagus, small intestine, colon, rectum, through to the mesentery, presenting a diagnostic dilemma. Due to the non-specific presentation, radiological, and endoscopic similarities between schwannomas and gastrointestinal stromal tumors (GISTs), a preoperative misdiagnosis is common. Recent improvements in immunohistochemistry have helped to define their biological identity based on widespread staining for S100/SOX10 with absence of KIT/DOG1 mutations. While techniques such as contrast-enhanced CT scans as well as endoscopic ultrasonography have certain diagnostic advantages, they are not sufficient to make a diagnosis without histopathological validation. Surgical resection is considered the mainstay of therapy, addressing both purposes. Endoscopic methods have been adopted for smaller lesions in more favorable locations, while surgical resection is considered standard for larger or more complex lesions.

**Conclusion:**

Despite their rarity, early and accurate diagnosis is important to avoid unnecessary interventions when conservative management may be appropriate. When treatment is required, resection strategies should be tailored to tumor characteristics.

## Introduction

Gastrointestinal (GI) schwannomas are rare, benign mesenchymal tumors that arise from Schwann nerve cells of the GI tract, but more classically from the myenteric Auerbach’s plexus [1]. They constitute a minority of all GI mesenchymal neoplasms and are most commonly found in the stomach [2, 3].

Unusual sites of occurrence present unique clinical and diagnostic challenges. While gastric schwannomas are well recognized, schwannomas that arise in unusual locations, i.e., the colon, rectum, esophagus, or mesentery, are frequently misdiagnosed, usually as gastrointestinal stromal tumors (GISTs) or other mesenchymal neoplasms [4, 5]. This diagnostic dilemma is due to several factors: imaging and endoscopic findings are often nonspecific and very similar to those of GIST [6, 7]. Preoperative diagnosis is rare in unusual locations due to the rarity of such tumors, subjecting them to overtreatment, and definitive diagnosis most often requires histopathological and immunohistochemical examination [7, 8]. Accurate diagnosis is important, as the therapeutic and prognostic implications are quite different from others gastrointestinal tumors. While schwannomas are removed locally with excellent results, GISTs may require adjuvant therapy and carry a risk of malignant transformation [2]. There is therefore a need for heightened awareness of the occurrence of schwannomas in uncommon GI sites, as well as the pitfalls of imaging and biopsy, to avoid misdiagnosis and to steer appropriate management.

This review aims to incorporate emerging developments in the pathogenesis of GI schwannomas at usual and unusual sites, with a focus on advances in diagnosis, evolving therapeutic strategies, and directions for further research.

## Epidemiology and Clinical Spectrum

After searching two databases (PubMed and Scopus) using the term “gastrointestinal schwannomas,” a total of 1,321 records were found. From these, we included all the studies focusing on the clinicopathological characteristics of gastrointestinal schwannomas across the gastrointestinal tract that comprised more than 20 patients, in order to provide a representative and clinically meaningful synthesis of larger series. The findings of large-scale institutional and multicenter trials (Table 1) showed that GI schwannomas occur most frequently in middle-aged to older adults with a median age of onset in the 5th and 6th decades of life [1, 2, 8–12]. However, their occurrence in pediatric populations is not excluded. At the same time, a consistent female predominance is characteristic, seen in almost all reports. The occurrence of GI schwannomas in the stomach appears to occur in more than half of cases, with an incidence rate exceeding 60%. The next most common location appears to be the intestine and esophagus. The most common locations in the stomach are the gastric corpus and antrum, and in the intestine, lesions occur most frequently in the colon, specifically in the ascending and sigmoid segments, and in the rectum [1, 2, 8–11]. Schwannomas of the small intestine, duodenum, appendix, pancreas, or mesentery are very rare and have been reported mainly in isolated case reports or small series [5, 11, 13].

**Table 1:** Clinopathological characteristics of studies on gastrointestinal schwannomas

Study ID	Participants (*n*)	Age/% Female	Anatomical Distribution (% of total)	Tumor Comorbidity	Average Tumor Size (cm)
Zhang et al., 2025[1]	229	56 (median)/64.6%	**Esophagus (10.0%):** 23• Unspecified: 3 (1.3%)• Upper: 5 (2.2%)• Middle: 6 (2.6%)• Lower: 9 (3.9%)**Stomach (76.9%)**: 176• Unspecified: 35 (15.3%)• Cardia: 3 (1.3%)• Fundus: 16 (7.0%)• Body: 94 (41.0%)• Angle: 3 (1.3%)• Antrum: 25 (10.9%)**Intestines (13.1%)**: 30• Duodenum: 2 (0.9%)• Ascending colon: 8 (3.5%)• Transverse colon: 6 (2.6%)• Descending colon: 3 (1.3%)• Sigmoid colon: 3 (1.3%)• Rectum: 8 (3.5%)	Gastrointestinal neoplasms: 3.4%•Hepatocellular carcinoma: 1.7%•Clear cell carcinoma (kidney): 1.3%	2.75 (range 0.3–9.5)
Peng et al., 2022[2]	78	52.1/70.5%	Esophagus: 17.7% (14/79) •Stomach: 68.4% (54/79) •Duodenum: 2.5% (2/79) •Colon: 5.1% (4/79) •Rectum: 2.5% (2/79) •Small intestine: 3.8% (3/79)	No comorbidities: 38.5%•Hypertension: 20.5%•T2DM: 6.4%•GI polyps: 12.8%•GI cancers: 6.4%	3.63 ± 2.03
Wu et al., 2020[8]	51	55.7 ± 11.4/62.7%	**Gastric body (51%) **: anterior wall (AW) 12 (23.5%), posterior wall (PW) 14 (27.5%) •**Gastric antrum (31%)** : AW 9 (17.6%), PW 7 (13.7%) •**Gastric fundus (8%)** : AW 2 (3.9%), PW 2 (3.9%) Esophagus: 2 (3.9%) Duodenum: 2 (3.9%) Rectum: 1 (2.0%)	–	**By site**: Gastric body – AW: 3.5 ± 1.7; PW: 4.9 ± 2.9Gastric antrum – AW: 3.0 ± 1.1; PW: 3.6 ± 2.2Gastric fundus – AW: 2.9 ± 2.3; PW: 6.3 ± 1.1Esophagus: 0.8 ± 0.8Duodenum: 2.2 ± 0.9Rectum: 10.3
Fan et al., 2024[9]	99	58.5/62.6%	**Gastric region (78.9%)**: Body: 45 (47.4%)• Antrum: 20 (21.1%)• Fundus: 10 (10.5%)**Other sites (21.1%):**Ascending colon: 5 (5.3%)•Small intestine: 4 (4.2%)•Duodenum: 3 (3.2%)•Esophagus: 2 (2.1%)•Esophagus + fundus: 1 (1.1%)•Sigmoid colon: 1 (1.1%)•Rectum: 1 (1.1%)•Ileocecal region: 1 (1.1%)•Mesogaster: 1 (1.1%)•Transverse mesocolon: 1 (1.1%)	–	4.04 (range 0.5–30)
Hou et al., 2006[10]	33	51.5 (median)/51.5%	Stomach: 24/33 (72.7%)• Esophagus: 4/33 (12.1%)• Colon: 2/33 (6.1%)• Rectum: 3/33 (9.1%)•		4.98 (mean) (range 1–12)
Singh et al., 2022[11]	44	59.2 (mean)/63.6%	Esophagus: 13.6% (6) • Stomach: 38.6% (17) • Small bowel: 13.6% (6) • Colorectal: 31.8% (14) • Pancreas: 2.3% (1)	Personal history of malignancy: 22.7% • CKD: 11.4% • COPD: 11.4% • CHF: 2.3% • Alcohol use: 25.0%	2.41 (overall mean, cm; site-specific: stomach 3.77, colorectal 2.41, esophagus 2.28, small bowel 2.10, pancreas 0.4)
Daimaru et al., 1998[12]	24	58 (mean)/62.5%	Stomach: 95.8% (23) • Colon: 4.2% (1) • Gastric distribution: middle third 54.2% • lower third 25.0% • upper third 16.7%	Malignancy: gastric carcinoma 20.8% • lung cancer 8.3% • cholelithiasis 8.3%	2.8 (range 0.5–7)

Reported tumor size varied widely, ranging from subcentimeter lesions to large masses exceeding 10 cm, with mean or median sizes generally between 2.5 and 5 cm, and with extreme values ​​from 0.3 cm to 30 cm [1, 9]. Gastric and colorectal schwannomas are usually well-circumscribed submucosal tumors discovered incidentally by imaging, endoscopy, or surgery for unrelated conditions. Larger or extraluminal lesions, however, tend to present with features of pressure or obstruction.

The clinical presentation of GIS varies, from asymptomatic incidental findings to symptomatic presentations based on a mucosal ulcer or mass. Incidental discovery in an asymptomatic patient remains the most common, accounting for 33%–53% of patients in large reports [2, 8, 9]. If a presentation occurs, it is usually nonspecific. The most common presenting symptoms are abdominal pain (16–37%), distension (10–20%), and discomfort or regurgitation (3–8%). Symptoms of gastrointestinal bleeding, such as melena or hematemesis, are rare (2–6%) and usually occur when the tumor has invaded the mucosa. Other rare symptoms include dysphagia with esophageal involvement, constipation or tenesmus with colorectal cancer, and unintentional weight loss in isolated cases [2, 8].

Epidemiological studies have also revealed considerable patterns of comorbidity. Type 2 diabetes mellitus and hypertension are the most frequent associated ailments, occurring in approximately 6% and 20% of cases, respectively [2]. Association with other gastrointestinal neoplasms or polyps in up to 10–12% of patients suggest a potential though unreferenced association with broader neoplastic predisposition [1, 2].

Site-specific studies also elucidate clinical heterogeneity. In the colon and rectum, the mean age is greater (61 years) with altered bowel habits, rectal bleeding, or occult blood positivity as the most common presentations. Sigmoid and cecal involvement are more common, and rectal schwannomas are rare but increasingly with the more extensive use of lower endoscopy [5].

## Pathogenesis and Molecular Biology

Current advances in molecular pathology have established that GI schwannomas are a biologically and genetically distinct subgroup of nerve sheath tumors [14]. The most significant observation has been the identification of highly recurrent in-frame insertions in the SOX10 gene, either within or near the high mobility group (HMG) box domain, in over 90% of the cases [15]. These mutations are very specific for GI schwannomas and rarely observed in non-GI schwannomas and other mesenchymal and melanocytic neoplasms, thus making a very good molecular diagnostic marker. Non-gastrointestinal schwannomas, on the other hand, typically possess NF2 mutations or SH3PXD2A::HTRA1 fusions [15–17].

SOX10-mutated GI schwannomas possess characteristic transcriptomic signatures in line with their well-established histomorphologic features, including microtrabecular Schwann cell morphology and lymphoid cuffing at the periphery. These are also associated with tumor size greater than mean, patient age greater than mean, and absence of fusion events [15]. Gastric and extra-gastric (e.g., colorectal, small bowel) schwannomas all have this SOX10-mediated pathway, suggesting there is a common pathogenesis between sites.

Immunohistochemistry remains the focus of GI schwannoma diagnosis. Diffuse SOX10 and S100 immunopositivity are diagnostic, with absence of CD117 (c-KIT) and DOG-1 being specific for differentiation of the tumors from GISTs. Vimentin and GFAP expression also establishes Schwann cell differentiation [1, 9, 18].

Histologically, GI schwannomas exhibit the characteristic Antoni A and Antoni B architectural patterns:

- Antoni A: Spindle cell-predominant regions with nuclear palisading and formation of Verocay bodies.
- Antoni B: Edematous, loosely cellular regions with intervening collagen fibers and myxoid stroma.

Approximately 60% of colonic schwannomas contain Antoni A or mixed A/B morphology. Mitoses are rare (mean 2.1/50 high-power fields), necrosis is uncommon, and lymph node metastasis is exceedingly rare, attesting to the benign biological behavior of the neoplasms [5, 19].

S100 protein was found to be almost universally positive (99% to 100%) in all series, making it the most sensitive diagnostic immunomarker for gastrointestinal schwannomas. SOX10, tested in more recent series, was also found to be very sensitive (97% to 100%). Vimentin was found to be consistently positive wherever it was tested. The positivity for CD34 was variable and was mostly focal (9% to 55%) [1, 2, 8–11].

Immunohistochemistry for markers associated with GIST, such as CD117 (c-KIT) and DOG1, was negative or rarely focally positive, with no mutation in KIT demonstrated when tested. Smooth muscle markers SMA and desmin were mainly negative but rarely focally positive. Expression for SDHB was maintained, while HMB45 was negative in the few studies that investigated these markers [1, 2, 8–10].

The proliferative indices were low in all cases, with Ki-67 indices less than 5–10%, although higher indices were seen in the malignant variants. Histologically, the tumors were composed of spindle cells arranged in sheets, fascicles, or trabecular patterns, which were usually surrounded by a cuff of lymphocytes. Antoni A/B regions and Verocay bodies were either absent or rudimentary in the majority of the GI schwannomas [1, 2, 8–10] (Table 2).

**Table 2:** Pathological and immunohistochemical findings of gastrointestinal schwannomas

Marker/Feature	Zhang et al., 2025[1]	Peng et al., 2022[2]	Wu et al., 2020[8]	Fan et al., 2024[9]	Singh et al., 2022[11]	Hou et al., 2006[10]	Daimaru et al., 1998[12]
S100	99.0% positive (207/209)	100% positive (78/78)	100% positive (51/51)	99% positive (98/99)	100% positive (43/43 tested; 1 NA)	100% positive	100% positive
SOX10	100% positive (64/64)	97.2% positive (35/36)	-	98% positive (97/98)	-	-	-
Vimentin	-	100% (32/32)	-	98% positive (97/98)	-	-	-
CD34	-	24.2% positive (18/74)	19.6% positive (10/51)	54.5% (54/99)	2.3% positive (1/44)	9.09% positive (3/33)	-
CD117 (c-KIT)	98.6% negative (3 focal +)	6.4% positive (5/78)	2.0% positive (1/50)	Negative, predominantly	-	100% negative	-
DOG-1	98.9% negative (2 focal +)	100% negative (68/68)	100% negative (45/45)	Negative, predominantly	-	-	-
SMA	91.2% negative (18/205 +)	17.9% positive (14/78)	2.0% positive (1/51)	Rare focal positivity	2.3% positive (1/44)	100% negative	100% negative
Desmin	91.9% negative (15/186 +)	-	4.4% positive (2/45)	Negative, predominantly	-	100% negative	100% negative
SDHB	100% positive (46/46)	-	-	-	-	-	-
HMB45	100% negative (27/27)	-	-	-	-	-	-
Ki-67 index	< 5% in 66.1%; 5–10% in 33.3%; >10% in 0.6%	1–10% in most (61/63)	median, 3%; range, 1%−60%	< 5% (benign), > 10% (rare, malignant)	Mean 3.0% (lesions > 2 cm; low proliferation)	-	-
Histological Pattern	Spindle-shaped cells with peripheral lymphoid cuff	-	-	Spindle morphology, featuring lymphatic sheath structures	Predominantly solid spindle-cell tumor with lymphoid cuff; rare mixed features, minimal necrosis	Spindle-cell tumor with lymphoid cuff; no Verocay bodies	Spindle-cell tumor with myxoid areas, nuclear palisading, and prominent peripheral lymphoid cuff

Unlike schwannomas, GISTs arise from the interstitial cells of Cajal and are diagnosed by activating KIT or PDGFRA mutations, which is the rationale for targeted therapy with tyrosine kinase inhibitors such as imatinib [20–23]. GISTs are immunohistochemically CD117 and DOG-1 positive but S100 and SOX10 negative. Other mesenchymal GI neoplasms, such as leiomyomas and NTRK- or GLI1-rearranged tumors, are characterized by distinct genetic and immunophenotypic features and reflect the molecular distinctiveness of GI schwannomas [24, 25].

## Diagnostic Approaches

### Imaging Modalities

**Fig. 1:**
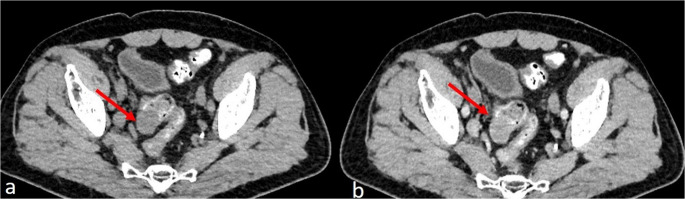
(**A**) Axial NECT scan showing a well-defined oval mass arising from the third part of the sigmoid colon (red arrow) (**B**) Axial CECT scan showing heterogeneous enhancement of the mass (red arrow)

**Fig. 2:**
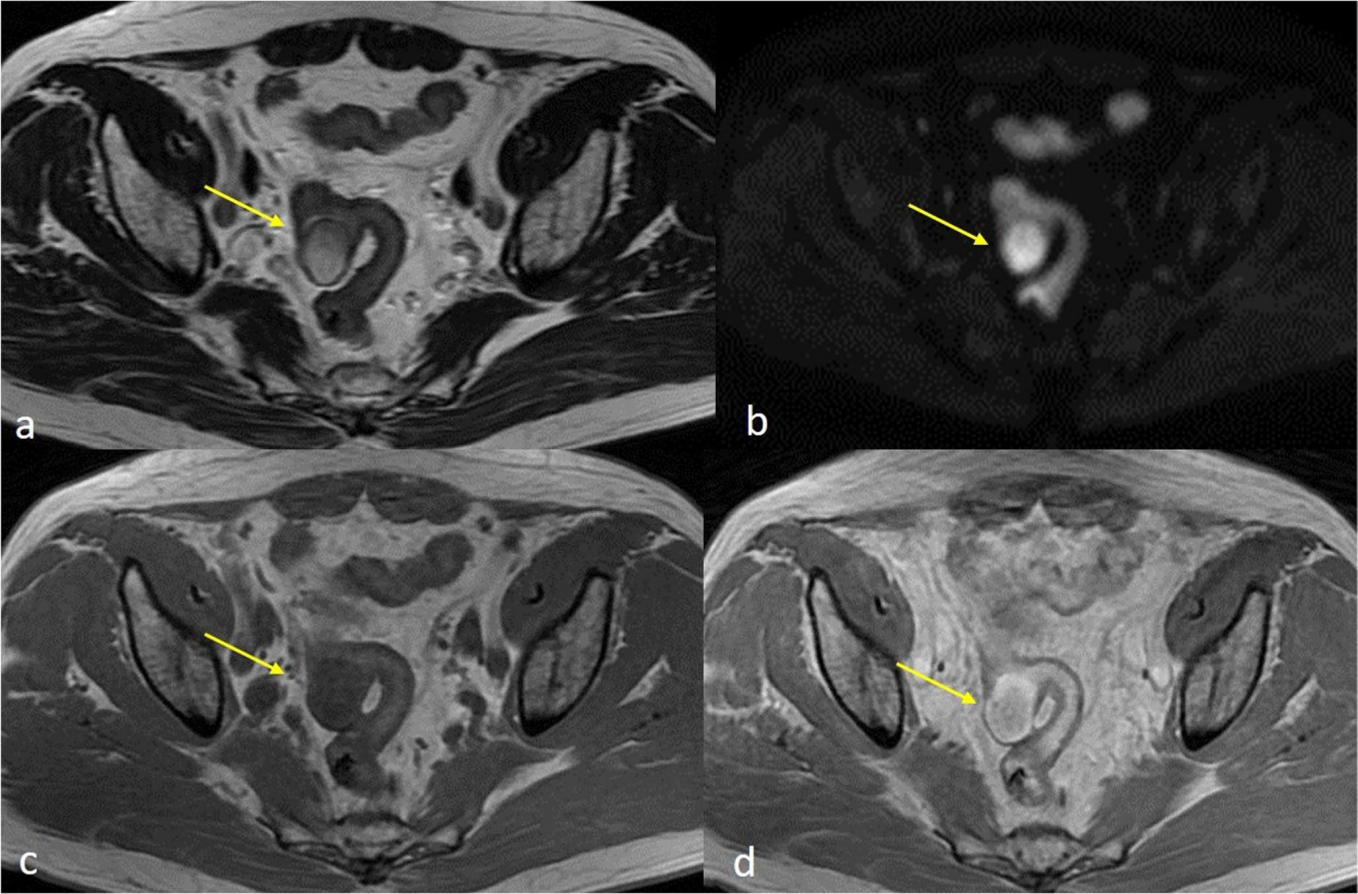
Abdominal MRI. (**A**) T2-weighted images show a hyperintense intramural sigmoid mass (yellow arrow) (**B**) with high B value in DW images (yellow arrow). (**C**) T1-weighted images before iv contrast administration show a hypointense intramural mass (yellow arrow) (**D**) T1WI post-contrast study, show heterogeneous enhancement of the mass. No lymphadenopathy was noticed (yellow arrow)

Contrast-enhanced CT is the primary imaging modality for GI schwannomas and is essential for differentiation from GISTs. Schwannomas typically present as small, well-defined, round or ovoid mural masses (Fig. 1a) with homogeneous attenuation and enhancement, reflecting the absence of necrosis, hemorrhage, or cystic degeneration [26–30]. In some cases, however, CECT scan may also show heterogeneous enhancement of the mass (Fig. 1b). There are characteristics that favor the diagnosis of schwannoma over GIST. These include smaller tumor size, smooth margins, homogeneous enhancement, lack of necrosis or cystic change, and the presence of regional or perigastric lymph nodes [26, 28–30]. A tumor size cutoff of 3.9 cm has been proposed to distinguish schwannomas from GISTs (AUC 0.808), with high specificity [26]. Quantitative CT analyses further demonstrate that schwannomas exhibit lower arterial-phase enhancement and normalized enhancement ratios compared with GISTs, consistent with lower tumor vascularity; an arterial enhancement degree of < 15.4 HU has been reported as a useful discriminator [28, 29]. In contrast to GISTs, metastasis and adjacent organ invasion are exceedingly rare in schwannomas [27]. MRI features are the same as with CT, with lesions typically isointense on T1-weighted and hyperintense on T2-weighted images and slightly post-contrast enhanced (Fig. 2).

### Radiomics, and Functional Imaging

Radiomics extracts high-dimensional features from CT to build discriminative models distinguishing between schwannomas and GISTs with AUCs of > 0.90, comparable to senior physicians [31, 32]. Combined models that integrate radiomics features with conventional CT parameters, such as tumor location and enhancement characteristics, managed to demonstrate superior diagnostic performance, with AUCs reaching 0.989, significantly outperforming traditional imaging models in differentiating schwannomas from GISTs, including tumors with varying malignant potential (*p* < 0.05) [31].

Fluorine-18 Fluorodeoxyglucose Positron Emission Tomography/Computed Tomography (18 F-FDG PET/CT), have limited specificity for schwannoma characterization and their findings vary considerably, requiring particularly careful interpretation. Specifically, in benign schwannoma mean SUVmax values ​​range from 4.1 ± 2.1 to 5.4 ± 2.7, with a substantial proportion of lesions demonstrating FDG uptake above commonly used malignant thresholds. A very important factor of FDG avidity is the location of the tumor with gastrointestinal and intra-abdominal schwannoma showing significantly higher SUVmax values ​​than those at other sites, which may be explained by the existence of peritumoral lymphoid cuffs, regional lymphadenopathy, and greater contrast enhancement on CT. Lesions with heterogeneous FDG uptake tend to have higher SUVmax values ​​and more frequent internal non-enhancing areas on MRI, features that may mimic malignant peripheral nerve sheath tumors despite benign histology [33, 34].

### Endoscopic and Endoscopic Ultrasound Findings

Endoscopic evaluation is often the first diagnostic step for submucosal gastrointestinal tumors. On routine endoscopy, gastrointestinal schwannomas typically appear as smooth, sessile submucosal masses with intact overlying mucosa, presenting as a well-circumscribed, homogeneous bulge; superficial ulceration or central depression may be observed in larger gastric or rectal lesions, but ulceration is generally uncommon [2, 35, 36]. These features are nonspecific and often indistinguishable from other subepithelial lesions, particularly GISTs [2, 35, 36].

EUS improves lesion characterization by defining the layer of origin and internal architecture. Schwannomas most commonly arise from the muscularis propria (fourth layer) and appear as well-demarcated hypoechoic lesions with homogeneous or mildly heterogeneous echotexture; marginal hypoechoic halos may be present, while cystic change, calcification, and significant internal vascularity are rare [36–39]. Although EUS enhances diagnostic confidence, it frequently fails to establish a definitive etiology [37–39]. When EUS-guided fine-needle aspiration or biopsy is performed, cytology may reveal spindle cells with abundant lymphocytes, reflecting the characteristic lymphoid cuff, and allows immunohistochemical differentiation from GISTs when adequate tissue is obtained [38, 40]. Nevertheless, these features are not pathognomonic, and histopathological and immunohistochemical confirmation remains essential for definitive diagnosis, consistent with current guideline recommendations [36, 41].

### Tissue Diagnosis and Adjunctive Approaches

Preoperative biopsy remains challenging since the tumors are intramural or submucosal. Endoscopic biopsies that are superficial yield non-diagnostic mucosa, with ultimate spindle cell recovery in only few cases [1, 8, 9]. EUS-FNA increases diagnostic yield and may identify spindle-shaped Schwann cells with a background of lymphocytes, opening the door to a possible diagnosis of schwannoma. Immunopanel testing (S100 and SOX10 positive; CD117 and DOG-1 negative) also serves to narrow preoperative differential diagnosis.

In contrast, EUS-guided fine needle biopsy (EUS-FNB) enables procurement of histologic core specimens, allowing preservation of tissue architecture and facilitating immunohistochemical analysis, which is mandatory for accurate diagnosis. In fact, EUS-FNB has been associated with higher sensitivity and accuracy compared to FNA, improved rates of adequate tissue acquisition for immunohistochemical, and fewer needle passes required to reach a diagnosis [42, 43]. Furthermore, FNB significantly increases the likelihood of obtaining material suitable for immunohistochemical staining, which is critical for distinguishing GIS from other mesenchymal tumors [44]. Although the use of rapid on-site evaluation (ROSE) may improve the diagnostic yield of FNA, current guidelines recommend EUS-FNB or EUS-FNA with ROSE, with a growing preference toward FNB given its superior histological yield [42].

Routine tumor markers and laboratory studies are of minimal utility in the diagnosis. CEA and CA19-9 levels are within normal limits, and routine hematologic or hepatic levels are not uniformly disturbed [8, 45].

### Diagnostic Difficulties and Surgical Confirmation

Despite all these developments, preoperative diagnosis remains limited to a minority of cases and in clinical practice, diagnosis is usually made postoperatively. Zhang et al. [1] showed that despite extensive use of CT/MRI, endoscopy, and EUS, only a small minority of cases were correctly suspected as schwannomas preoperatively, with most lesions labeled as GISTs, submucosal tumors, or nonspecific masses. Even EUS-guided fine-needle aspiration was rarely diagnostic, and characteristic features such as peritumoral lymphadenopathy were infrequent and nonspecific. Previous studies [8, 9, 46] also confirm this diagnostic difficulty, as CT and endoscopy typically demonstrated nonspecific submucosal or muscularis propria lesions, and endoscopic biopsies were often nondiagnostic due to superficial sampling. Schwannomas were frequently discovered incidentally during surgery or misclassified as GISTs, neuroendocrine tumors, or other malignancies, with definitive diagnosis achieved only after resection.

## Therapeutic Strategies

GI schwannomas is a slow-growing neoplasm with a low probability of malignant transformation. Therefore, a conservative approach (observation/surveillance) is a viable strategy, especially for small, asymptomatic, tissue-confirmed lesions [1, 2, 4]. The ACG Clinical Guideline on Gastrointestinal Subepithelial Lesions (SELs) states that asymptomatic benign SELs do not require resection, and lesions with very low risk for malignant transformation can be managed on a case-by-case basis considering size, resection risk, and overall patient health [42]. The AGA Clinical Practice Update recommends that for gastric SELs arising from the muscularis propria that are < 2 cm, surveillance using EUS should be considered [41]. ASGE similarly suggests surveillance EUS for gastric subepithelial tumors < 2 cm without high-risk features [47]. This approach is even safer in tissue-confirmed schwannomas in which the rationale for resection is primarily symptom-driven rather than oncologic. Ho et al., showed that for small (< 2 cm), asymptomatic, homogeneous-appearing SELs from the muscularis propria without high-risk EUS features, no lesion-related adverse outcomes occurred within the first 3 years and the surveillance interval may be safely extended to every 2 years for micro-SELs (< 10 mm) [48]. The ASGE has proposed 6- to 12-month EUS surveillance intervals for small GISTs without high-risk features, although this is more conservative than what may be needed for confirmed schwannomas [47]. Finally, for confirmed schwannomas, a reasonable approach includes periodic EUS to monitor for size change, with resection reserved for symptomatic progression, rapid growth (> 20% per year), or development of high-risk EUS features (irregular borders, cystic spaces, heterogeneity) [49, 50].

When GI schwannomas require intervention, they are treated surgically as systemic or targeted medical therapies are lacking. Resection should be considered in symptomatic tumors [41], increase in size on serial imaging, particularly rapid growth (> 20% per year) [49], larger lesions (commonly > 2–3 cm) [41, 47], especially when diagnosis is uncertain, development of high-risk features on EUS (irregular borders, heterogeneity, cystic changes, ulceration) [41, 42], as well as in patient preference for resection over lifelong surveillance [42]. Treatment is therefore based on complete local excision with histologically negative margins (R0 resection) [49, 51]. This resection can, however, be achieved by a variety of approaches, either open surgery or minimally invasive methods, with the optimal approach being complete surgical excision with negative margins, achieved by wedge resection, segmental resection or enucleation, depending on the size of the tumor, anatomical location and proximity to critical structures.

Gastric schwannomas are most commonly treated with wedge resection, while larger or deeply located lesions may require segmental or partial gastrectomy [52]. Esophageal schwannomas are usually treated with enucleation, which is increasingly performed via minimally invasive thoracoscopic or laparoscopic techniques, which reduce morbidity and hospitalization [51, 53]. Lymphadenectomy is not indicated, as nodal metastasis is exceedingly rare [51, 53]. Open surgical resection achieves high R0 rates and excellent long-term disease control. Postoperative recurrence is exceptionally rare, and routine surveillance is generally unnecessary following complete resection [1, 2, 8].

In appropriately selected cases, minimally invasive surgery has become the preferred strategy. Laparoscopic and thoracoscopic resections achieve similar R0 rates and complication rates compared to open surgery, but with shorter surgical times, less blood loss, and lower costs [1–3]. Endoscopic resection, including endoscopic submucosal dissection (ESD) and endoscopic full-thickness resection (EFTR), is a safe and effective alternative for small (< 3 cm), well-circumscribed, noninvasive lesions, especially in the stomach [1, 2, 54–56]. Complete resection rates range from 85 to 88%, with no recurrence or metastasis reported during long-term follow-up (mean 21–37 months, extending up to 140 months) [54–56].

Comparative studies show similar recurrence and complication rates between minimally invasive and open resection, but with reduced operative time, less intraoperative blood loss, and lower operation cost, without significant differences in hospital stay or adverse events [2, 8, 55, 56].

**Fig. 3:**
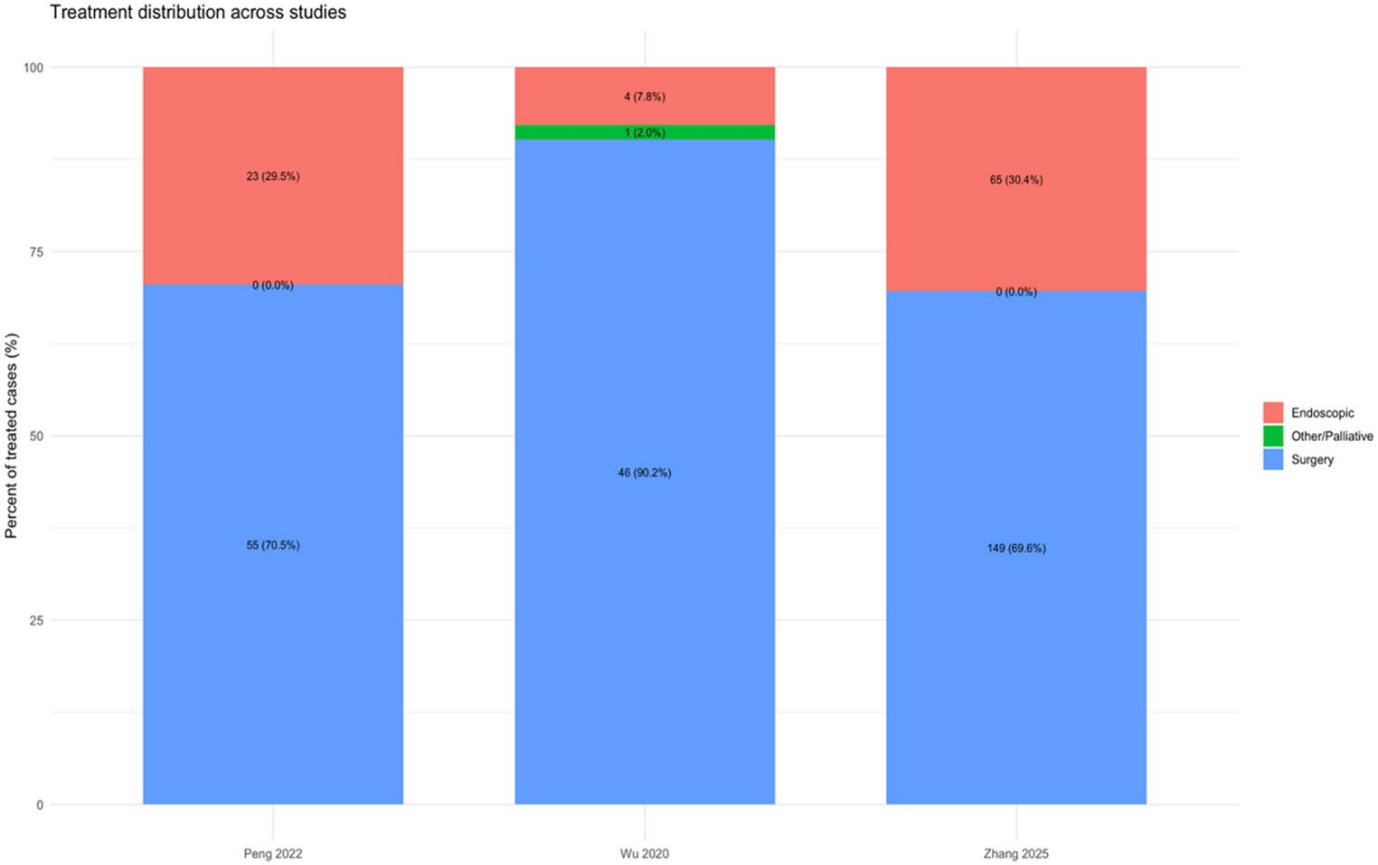
Treatment distribution across studies. Stacked bar chart showing the proportion of endoscopic, surgical, and other/palliative treatments in major published series. Surgery remains the predominant modality, while endoscopic resection accounts for approximately one-third of cases in recent cohorts, reflecting increased utilization over time

Throughout the contemporary series, the choice of treatment modality for gastrointestinal schwannomas has always been dependent on the tumor size and its location. In the largest series to date, Zhang et al. [1] showed that while the percentage of patients treated endoscopically was around 30%, the majority were still treated surgically (around 70%) (Fig. 3). The tumor size was also smaller in the endoscopic group compared with the surgical group (1.60 cm vs. 3.50 cm, *P* < 0.001), and there were no differences in age between the two groups. It is also important to note that the percentage of patients treated with endoscopic resection has been steadily increasing with time due to improved expertise and the use of advanced techniques.

**Fig. 4:**
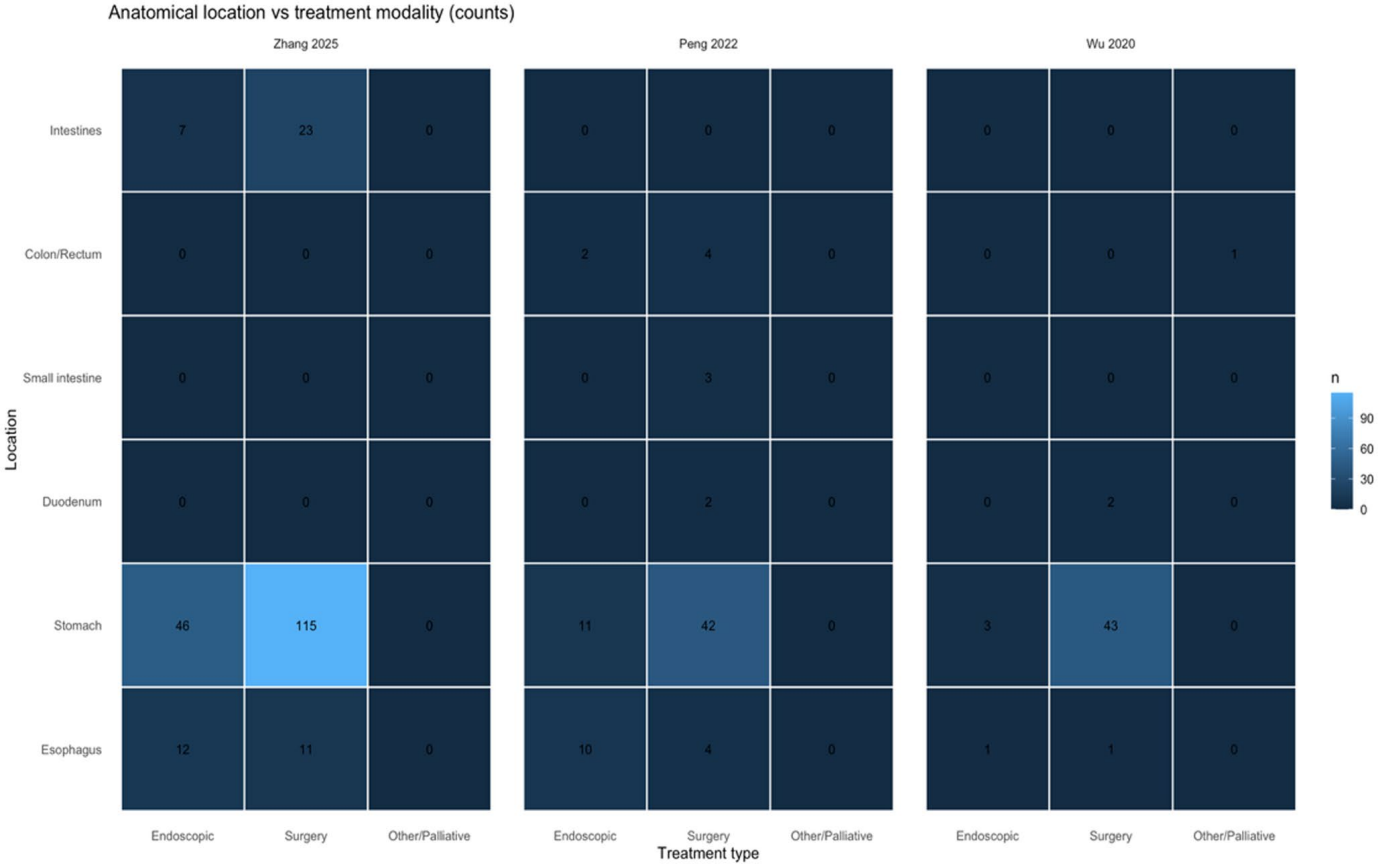
Anatomical location versus treatment modality. Heatmap illustrating the relationship between tumor location and treatment approach across studies. Endoscopic resection is more frequently used for gastric and esophageal schwannomas, whereas intestinal and colorectal locations are more commonly managed surgically, highlighting the influence of anatomical site on therapeutic decision-making

This size-dependent selection is further supported by the work of Peng et al. [2], who found a preference for endoscopic resection in smaller tumors (approximately 1.9 cm) and a higher probability of surgery in larger tumors (approximately 4.4 cm). Endoscopic therapy resulted in shorter hospital stay and lower cost with no increased risk of recurrence. There was also a dependence on the location of the tumor, with gastric and esophageal tumors being more amenable to endoscopic therapy and intestinal and colorectal tumors being more amenable to surgery (Fig. 4). This dependence on tumor location in the selection of treatment is well depicted in the work of Wu et al. [8], in which the major type of surgery performed is laparoscopy, with endoscopic resection being performed in selected patients only.

The overall morbidity was low and seemed procedure-related rather than tumor-related. The incidence of complications was similar for both endoscopic and surgical modalities, and there was no procedure-related mortality. The long-term results were excellent for the benign schwannomas, tending towards a complete cure following resection.

Taken together, these results highlight the importance of the fact that the choice of management is not merely between endoscopy and surgery but rather individualized based on the size, location, depth of origin, and expertise available, with the goal of resection providing both diagnosis and cure in the majority of patients with benign conditions.

To date, no effective medical or targeted therapies exist for gastrointestinal schwannomas. As these tumors lack KIT or PDGFRA mutations, tyrosine kinase inhibitors (e.g., imatinib, sunitinib, bevacizumab) are ineffective and should not be used [6]. Experimental pharmacologic agents, such as brigatinib and recombinant human neuregulin-1β1, have shown limited benefit in NF2-related schwannomatosis, but their role in sporadic GI schwannomas remains unproven and investigational [57, 58]. Current management therefore remains purely surgical. Preclinical studies employing bacterial immunotherapy and checkpoint blockade have been promising in model systems of schwannoma, but this has not been applied to gastrointestinal disease and remains speculative [59].

## Conclusion

Gastrointestinal schwannomas are uncommon but clinically significant tumors that are biologically and prognostically distinct from other gastrointestinal mesenchymal neoplasms. Although progress has been made in the areas of imaging and endoscopy, the preoperative correct diagnosis remains difficult, especially for nongastric primaries, and is ultimately made based on the results of postoperative histopathological and immunohistological analysis. The procedure of complete resection is both diagnostic and therapeutic for the vast majority of patients with the benign variant and offers excellent long-term results. Personalized treatment strategy, considering the size and location of the schwannoma, as well as the site and level of origin, supports the growing interest in the endoscopic resection for selected patients, while surgery remains the mainstay for the treatment of larger and more complex tumors.

## Data Availability

No datasets were generated or analysed during the current study.
